# 3D Rapid Prototyping for Otolaryngology—Head and Neck Surgery: Applications in Image-Guidance, Surgical Simulation and Patient-Specific Modeling

**DOI:** 10.1371/journal.pone.0136370

**Published:** 2015-09-02

**Authors:** Harley H. L. Chan, Jeffrey H. Siewerdsen, Allan Vescan, Michael J. Daly, Eitan Prisman, Jonathan C. Irish

**Affiliations:** 1 TECHNA Institute, University Health Network, Toronto, Ontario, Canada; 2 Department of Surgical Oncology, University Health Network, Toronto, Ontario, Canada; 3 Institute of Medical Science, University of Toronto, Toronto, Ontario, Canada; 4 Department of Biomedical Engineering, Johns Hopkins University, Baltimore, Maryland, United States of America; 5 Department of Otolaryngology–Head & Neck Surgery, University of Toronto, Toronto, Ontario, Canada; 6 Department of Otolaryngology, Mount Sinai Hospital, Toronto, Ontario, Canada; 7 Vancouver General Hospital, University of British Columbia, Vancouver, British Columbia; University of Southern California, UNITED STATES

## Abstract

The aim of this study was to demonstrate the role of advanced fabrication technology across a broad spectrum of head and neck surgical procedures, including applications in endoscopic sinus surgery, skull base surgery, and maxillofacial reconstruction. The initial case studies demonstrated three applications of rapid prototyping technology are in head and neck surgery: i) a mono-material paranasal sinus phantom for endoscopy training ii) a multi-material skull base simulator and iii) 3D patient-specific mandible templates. Digital processing of these phantoms is based on real patient or cadaveric 3D images such as CT or MRI data. Three endoscopic sinus surgeons examined the realism of the endoscopist training phantom. One experienced endoscopic skull base surgeon conducted advanced sinus procedures on the high-fidelity multi-material skull base simulator. Ten patients participated in a prospective clinical study examining patient-specific modeling for mandibular reconstructive surgery. Qualitative feedback to assess the realism of the endoscopy training phantom and high-fidelity multi-material phantom was acquired. Conformance comparisons using assessments from the blinded reconstructive surgeons measured the geometric performance between intra-operative and pre-operative reconstruction mandible plates. Both the endoscopy training phantom and the high-fidelity multi-material phantom received positive feedback on the realistic structure of the phantom models. Results suggested further improvement on the soft tissue structure of the phantom models is necessary. In the patient-specific mandible template study, the pre-operative plates were judged by two blinded surgeons as providing optimal conformance in 7 out of 10 cases. No statistical differences were found in plate fabrication time and conformance, with pre-operative plating providing the advantage of reducing time spent in the operation room. The applicability of common model design and fabrication techniques across a variety of otolaryngological sub-specialties suggests an emerging role for rapid prototyping technology in surgical education, procedure simulation, and clinical practice.

## Introduction

Surgical procedures in otolaryngology-head and neck surgery can pose considerable challenges even to the most experienced surgeons during resection of infiltrative diseases and reconstruction of anatomical structures. These challenges arise from performing excision of lesions within critical and anatomically complex structures in the head and neck region. For example, endoscopic skull base surgery for pituitary gland resection or clivus ablation involves dissection in close proximity to the surrounding optic nerve and carotid arteries. The risk of surgical complications is increased if surgeons are unable to maintain adequate visualization from the endoscope. Surgical proficiency typically requires a long training process from apprenticeship under senior surgeon supervision and/or cadaveric dissection. Many approaches are under investigation to improve the efficiency of surgical skills learning, while maintaining the overall quality. Snyderman et al. proposed a structural training program that allows the learner to incrementally acquire surgical skills in five stages [1]. Additionally, the availability of image-guided surgery (IGS) systems in clinical practice are also now an integral component of surgical skill development [2]. Virtual reality (VR) systems for the surgical simulation have shown promising outcomes [3–13], however, they are generally expensive and provide limited haptic feedback. Furthermore, they are limited to use virtual instrument and real surgical instruments not available in surgical simulation [14].

Development of surgical guidance systems also presents challenges of limited realistic environments during iterative system performance testing prior to deployment in the operating room. For example, emerging image-guided technologies such as intra-operative CBCT [15], 3D ultrasound [16] and interventional MRI [17] have all undergone extensive pre-clinical evaluation prior to clinical implementation.

Traditional approaches to the development of surgical competency and image-guidance technology involve the use of biological specimens in a pre-clinical setting. However, the use of cadaveric specimens requires considerations of resource availability and bio-safety factors. In contrast, animal studies raise ethical issues and may not provide a realistic representation of human anatomy. In both scenarios, there are costs associated with specimen transport and storage.

The need for realistic and reproducible clinical environments for surgical training, simulation and image-guidance system development motivates the design and fabrication of realistic anatomical phantoms. The development of realistic 3D phantom models for head and neck surgery has the potential to provide a threefold advantage: 1) from an educational perspective, it provides realistic and customizable environments for surgical trainees; 2) from a surgical perspective, it enables the fabrication of patient-specific models for surgical planning and procedure simulation before embarking on the actual surgery; and 3) from a research perspective, it facilitates technology development in an environment that mimics clinical practice.

This manuscript reports the development of a set of high-fidelity surgical models using rapid prototyping technology for endoscopic sinus surgical training, simulation of advanced skull base surgery, and patient-specific mandible plating. Additionally, we used these phantoms to facilitate the development and clinical implementation of an endoscopic augmented reality image-guidance system. Recent advances in rapid prototyping and 3D printing technology [18–23] have enabled the fabrication of realistic 3D models of head and neck anatomy using materials simulating anatomical properties of tissues.[24] Three rapid prototyping head and neck models were designed and fabricated for use in this translational research process, including: i) an endoscopic sinus phantom for training endoscopists and facilitating quantitative evaluation of methods for surgical tool tracking and registration of endoscopic video with CBCT imaging; ii) a high-fidelity sinus and skull base phantom with bone and soft-tissue features in combination; and iii) clinical implementation in the operating room using patient-specific mandible templates for mandibular reconstruction.

The structure of this manuscript as following: Section 2 provides literature review of existing phantom and physical model available for head and neck surgical application. Section 3 presents methods and materials for the fabrication of the three 3D head and neck models. Section 4 presents applications of these custom 3D models in endoscopy-enhanced image-guidance system development, surgical simulation and training, and reconstruction surgery in the operating room. Section 5 summarizes the technology development and surgical applications, and discusses the role of rapid prototyping in the advancement of surgical guidance systems from bench-top to clinical practice.

## Background

Early development of tangible models for endoscopic sinus surgery (ESS) simulation originated from Yamashita et al. in 2004 [25]. The main core of their sinus model consisted of 5 components including: bone slice left/right side, bone slice left/right ethmoid, and bone slice nasal septum. All components were made with plastic. Chen and Ling reported on their experiences using this phantom for training in endonasal surgical procedures [26]. Comments from the users in training were positive on the realistically reproduced nasal bone structure compared with a cadaver model and real patients. This phantom is commercially available through the SurgTrainer (Tsukuba City, Ibaraki, Japan). The cost of the main components is $2,000 USD.

More recently, Nogueira et al. reported on an endoscopic sinus and skull-base surgery simulator. This model was named SIMONT (Sinus Model Oto-Rhino Neuro Trainer, Recife, Brazil) which was fabricated with two special materials that simulate bony and soft tissue [27]. A resin material was used to simulate human bone, including structures of the frontal sinus and recess, frontal beak, partial ethmoid cells, opening for the placement of the maxillary sinus and lamina papyracea, and anterior sphenoid wall. The bony lateral nasal wall was laminated by a custom material named Neoderma (Pro Delphus, Recife, Brazil) that simulates mucosal properties. The septum, nasal turbinates, uncinate process, and other soft-tissue structures were fabricated separately with special resin and Neoderma material. The fabrication processing and the material compound were not disclosed in their publication. Filho et. al. [14] and Zymberg et. al [28] also reported using Neoderma custom material to build neuroendoscopic training models. The cost of the SIMONT was ~$400-$1,000USD [22]. Ossowski et al [29] developed a nasal model (NM) for teaching endoscopic nasal skills in scoping procedures. The randomized blinded control trial showed that medical students trained with NM had an improvement in procedure performance in terms of time compared to non-trained students. The trained students also demonstrated a reduction in subjective discomfort during endoscopic procedures in patients. The fabrication details of the NM were not mentioned in the publication.

Bichlmeier et al. proposed the true-scale of the Visible Korean Human Phantom to provide a realistic environment for the development and evaluation of medical augmented reality technology [30]. However, the scope of this phantom was designed to facilitate the development of external camera video augmentation which may not be applicable for endoscopic applications.

Rapid prototyping technology has found clinical applications in mandible and cranial surgery for surgical planning of the reconstruction site [21, 31–51]. In mandible reconstruction surgery, the challenge arises from maintaining the functional aspect of the structure in mastication, speech, swallowing and tongue base support for the airway while achieving the best possible aesthetic satisfaction. Both functional and aesthetic outcomes are relevant to the shape and position of the bone grafts which directly influence the geometric correlation between maxilla and mandible, and the position of the condyle in the glenoid fossa. Connecting the bone grafts to the structures of the recipient site typically uses titanium plates which require intra-operative fabrication. To facilitate preoperative surgical planning, research had been proposed using a patient-specific rapid prototyping model created from preoperative CT, as a template for the reconstruction plate fabrication.

## Materials and Methods

This section provides methods and fabrication processes of rapid prototype models for three applications of Head and Neck surgery. Section 3.1 describes the creation of a sinus phantom for endoscopic surgical training and advanced image-guided surgery system development. Section 3.2 provides the detail of a multi-material phantom for advanced skull base surgical simulation. Section 3.3 describes implementation of patient-specific mandible templates for surgical reconstruction. All studies are approved by our institute University Health Network Research Ethic Board. Written consent is acquired from each participant in the reconstructive surgery study by research coordinator during surgery pre-admission visit.

### Sinus Phantom for Endoscopic Surgical Training and Advanced Image guided Surgery System Development

#### Phantom Requirements

To provide a realistic clinical environment for surgeons training in endoscopic surgery and to facilitate the development of advanced IGS system, a custom endonasal phantom was designed using rapid prototyping techniques. The criteria for phantom design includes: 1) presentation of realistic anatomical structures; 2) durability for repeat lab usage; 3) identifiable features for endoscopic visualization; 4) rigid landmarks for precise pointer localization; 5) nasal pathways wide enough for surgical tool and endoscope access; and 6) a rigid, homogeneous material with comparable CT (Hounsfield) number to bone (1000-3000HU).

#### Phantom Design

The endoscopic phantom was designed using a high-resolution CT ((0.8 × 0.8 × 1.25) mm^3^ voxel size) of a cadaveric head under institutional research ethic board approval. The CT scanner employed for image acquisition is GE Lightspeed multislice (16-Slice) scanner (GE Healthcare, Massachusetts). The selected specimen exhibited well pneumatized paranasal sinuses and limited evidence of inflammatory changes. The selection criteria also considered the native tissue architecture for the requirement of digital processing modifications. Specimens with deviated septum, inflammation or swelling of the lining of the nasal passages and sinuses were not selected because it would create narrowing of the nasal corridor and thus inhibit the ability to insert instrumentation into the phantom.

**Fig 1:**
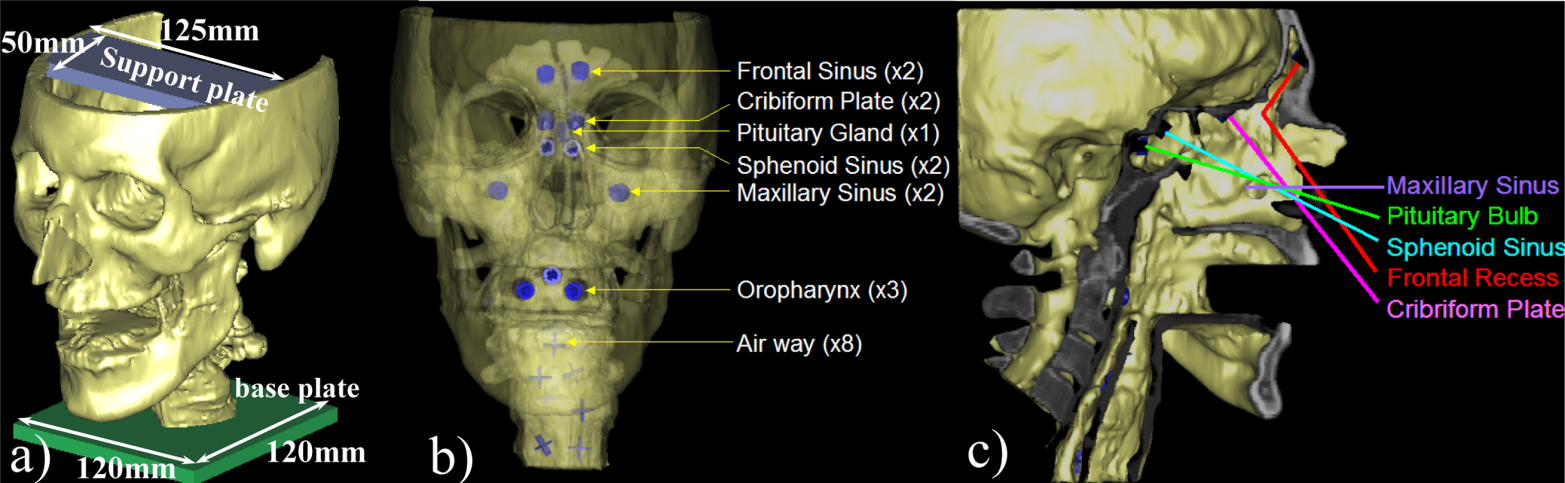
Illustration of the endoscopic navigation head and neck phantom. (a) Computer model of the final phantom design. (b) Semi-transparent view showing fiducial marker locations in the phantom. (c) Section view showing possible trajectories for surgical instruments.

The phantom creation procedure started with importing the CT image into the 3D visualization software (Mimics v12.0, Materialise, Ann Arbor MI) for digital processing. The coarse paranasal structure was first extracted by global thresholding (soft tissue threshold value -800HU to 100HU and bone tissue threshold value 100-3071HU) and region growing on the volumetric image. Slice-by-slice manual contouring was then applied in orthogonal tri-planar views to edit and detail the fine structures. Anatomy defined as rigid structures included the frontal, maxillary, and sphenoid sinuses, nasal bones, maxilla, orbital walls, palate, and skull base. The septum was modeled as a bony structure to provide separation between the left and right nasal sinus. The cartilaginous tissues including the ethmoid sinuses and nasal turbinates (superior, middle, inferior) were discarded from the structure to allow surgical instruments to gain access and manipulate in the nasal pathway. Round window openings were created on the anterior wall of the sphenoid sinus and the lateral wall of the maxillary sinus for endoscopic examination. For use with flexible endoscopic devices (e.g., laryngoscope), the air pathway was manually delineated where the larynx and pharynx are combined into a common channel. A support plate was added on the cranium region to enable mounting of a navigation reference tool, and a base plate (120 mm x 120 mm) was attached to the laryngopharynx to act as a podium for the phantom (Fig 1A).

**Fig 2:**
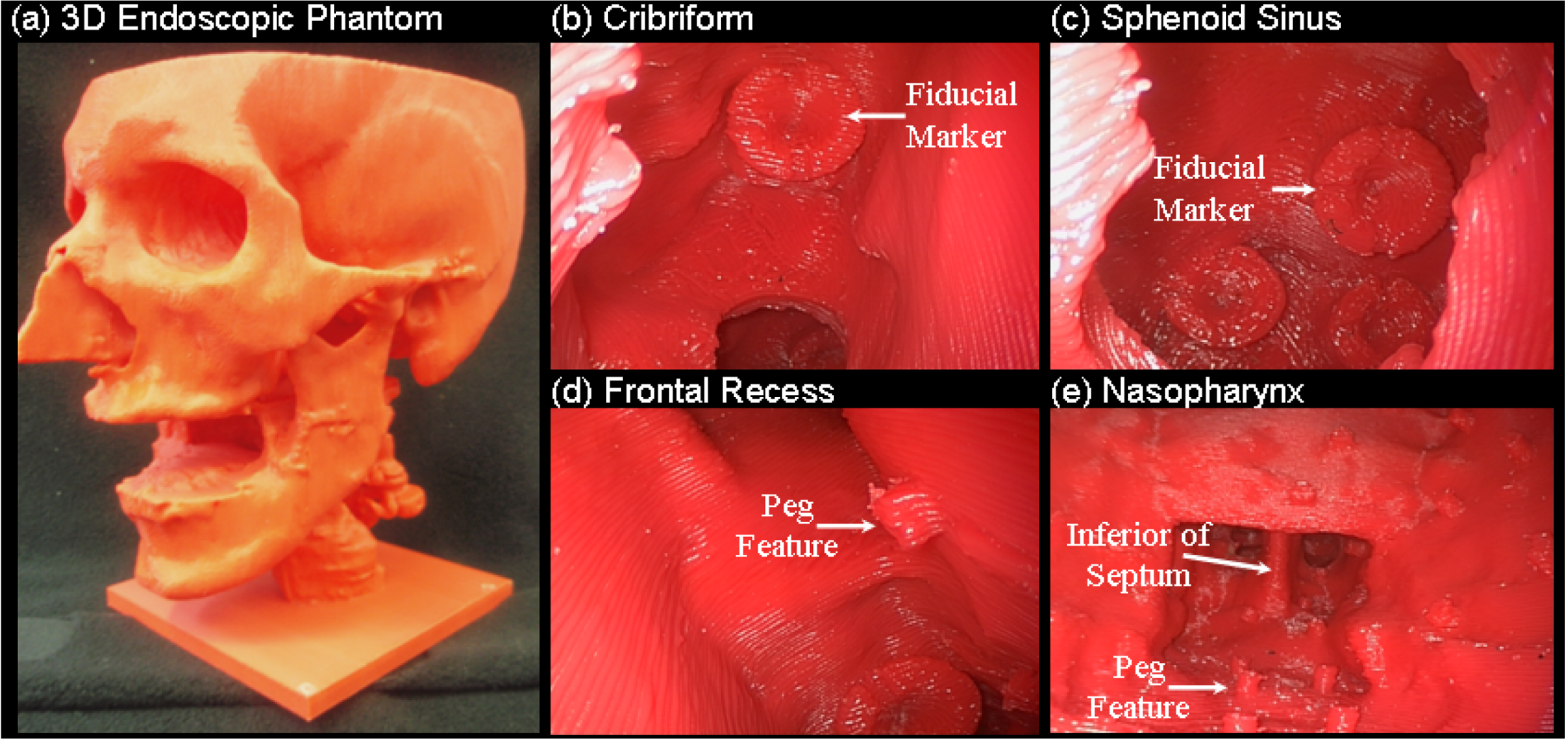
Photographs of 3D endoscopic phantom for the development of real-time tracking/navigation and image fusion methods. (a) The resulting phantom printed with ABS material. Endoscopic images of: (b) the fiducial marker on right cribriform plate; (c) the fiducial marker at the posterior aspect of the sphenoid sinus; d) the peg feature in the frontal recess; and (e) a view of the nasopharynx.

Target registration error (TRE) and fiducial registration error (FRE)[52] measurements with pointer localization is a common approach to assess tracking system accuracy. To facilitate these measurements, rigid landmarks (Fig 2C) in the form of cylindrical fiducial markers (8 mm diameter, 5 mm height) were introduced at anatomically relevant locations in the model. Cone-shaped divots (5 mm diameter, 4 mm height) were then removed from each cylinder along its central axis to provide precise points that can be localized in CT and with a tracked pointer. On the top surface of the marker, a 1 mm wide t-shaped notch was imprinted to a depth of 1mm. This notch provides an indication of the relative orientation of the endoscopic perspective view on the anatomy. Fiducial markers were placed at the left/right frontal sinus (x1 each side), cribriform Plate (x2), pituitary gland (x1), sphenoid sinus (x2), left/right maxillary sinus (x1 each side), and oropharynx (x3).

To enhance the anatomical features of the model, 80 cylindrical pegs (Fig 2E) (1 mm diameter, 3mm height) were artificially introduced on the left side of the phantom, including all sinus areas and the nasal and oral cavities. For use with flexible endoscopes, 8 cross-marks with 1mm width were engraved on the throat airway wall. Although not anatomically realistic, these features provide well-defined details that can be used to evaluate image fusion methods (e.g., endoscopy-CT registration). The features and markers described above were designed using a combination of Boolean operations (e.g., union, subtraction, intersection) within the design software in order to assemble them on the segmented skull model. Fig 1 illustrates the final design of the endoscopic sinus phantom.

#### 3D Fabrication

The rapid prototyping machine employed in printing was Vantage (Stratasys Inc. Eden Prairie MN), which is based on fused-deposition modeling (FDM) technology with the capability to build layers with thickness over the range of 0.127–0.33mm. For durability and reusability, acrylonitrile butadiene styrene (ABS) was selected as the fabrication material. The 3D physical model of the endoscopic sinus phantom is illustrated in Fig 2. The fabrication process took 50 hours including prototyping and support material dissolution. The cost of the phantom was ~$600 USD with an in-house prototype machine.

### Multi-Material Phantom for Advanced Skull Base Surgical Simulation

#### Phantom Requirements

The main challenge in the fabrication of a multi-material phantom for surgical simulation is to mimic the complex 3D structure of the paranasal sinuses and skull base with realistic tissue properties compatible with current rapid prototyping technology. Such a phantom involves simulated bony and soft tissue materials that demonstrate accurate density (CT Hounsfield number) and anatomically realistic flexibility, cut-ability, and drilling purchase to provide surgical tactile feedback similar to clinical dissection.

#### Phantom Design

The phantom design considers the combination of two distinct types of materials to simulate the compounds of bony and soft tissue anatomical structures. The same CT image as described in Section 3.1 has been used for developing this surgical simulation phantom. Structures of interest were segmented using a combination of automatic and semi-automatic intensity thresholding, region growing, and slice-by-slice manual contouring in orthogonal tri-planar views. The bony structures defined in the phantom design include the frontal, ethmoid, maxillary, and sphenoid sinuses, the nasal bones, maxilla, orbital walls, palate, and skull base. These bony structures required material properties similar to bone (for appropriate x-ray attenuation) and realistic fracture characteristics and drilling purchase. The soft tissue structures include the septum, middle turbinates, and inferior turbinates. Soft tissue components were designed to be pliable and non-brittle, and to allow excision with surgical tactile feedback using common surgical instruments, such as forceps and through-cuts.

To combine the bony and soft tissue structures, the turbinates were modified to include ball joints so that they could be snapped into complementary holes defined in the surrounding bony material. Similarly, the septum was modified to include slot joints along the top and bottom edges of the structure to allow the septum to be slid into the phantom in the final assembly.

#### 3D Fabrication

To compose a multi-material 3D model, the anatomical structures described above were first segmented as individual components. The bony structures (e.g., skull base, maxilla, palate and clivus) were joined together as a contiguous object. Soft-tissue structures were segmented into five individual objects that consisted of the septum, left/right middle turbinates, and left/right inferior turbinates. The individual components were printed separately using appropriate materials to simulate their anatomical properties. The phantom materials needed to demonstrate realistic physical, tactile, and visual characteristics comparable to the simulated anatomy. Also, they needed to satisfy requirements from both a surgical perspective (e.g., drilling purchase, flexibility and cut-ability) and a medical imaging perspective (e.g., attenuation coefficient or Hounsfield Units for CT, or proton density or water content for MRI). Although existing rapid prototyping technology is capable of fabricating multi-material objects, the most commonly available materials (e.g., polycarbonate, powder plaster, rubber) do not immediately yield compounds that satisfy the requirements for the bone and soft-tissue tissues. This limitation raised a challenge for material selection and post-processing techniques during the early stages of prototype development.

Plaster powder was selected as the base material for initial investigation, as it offers different types of composites and post-processing techniques which can provide flexibility and elasticity in the final material properties. To select appropriate materials, a series of sample objects fabricated from a variety of powders in combination with a selection of post-processing techniques was created. The form of the sample object consisted of a honeycomb associated with thin hexagonal walls and a solid rectangular base plate. In the material selection, an expert sinus surgeon qualitatively evaluated drilling purchase on the base plate and cut-ability on the hexagonal walls. This evaluation was conducted with standard surgical instruments so that the surgeon could sense the differences between materials, and compare the tactile feedback to those experienced during clinical practice. Drilling purchase tests were conducted with a 4 mm diamond bur and a 3 mm cutting bur in an otologic drill (Medtronic Xomed, Jacksonville FL), and cutting of the materials was tested with standard surgical equipment (e.g., Kerrison rongeurs and through-cutting instruments, Karl Storz, Tuttlingen Germany). The materials evaluated as closest to mimicking native sinus tissue air cells by the surgeon were selected. The chosen material for the bony structure was a ZP-130 plaster powder (ZCorp, Burlington, MA) with post-processing by 15-minute infiltration by CA101 cyanoacrolate.

**Fig 3:**
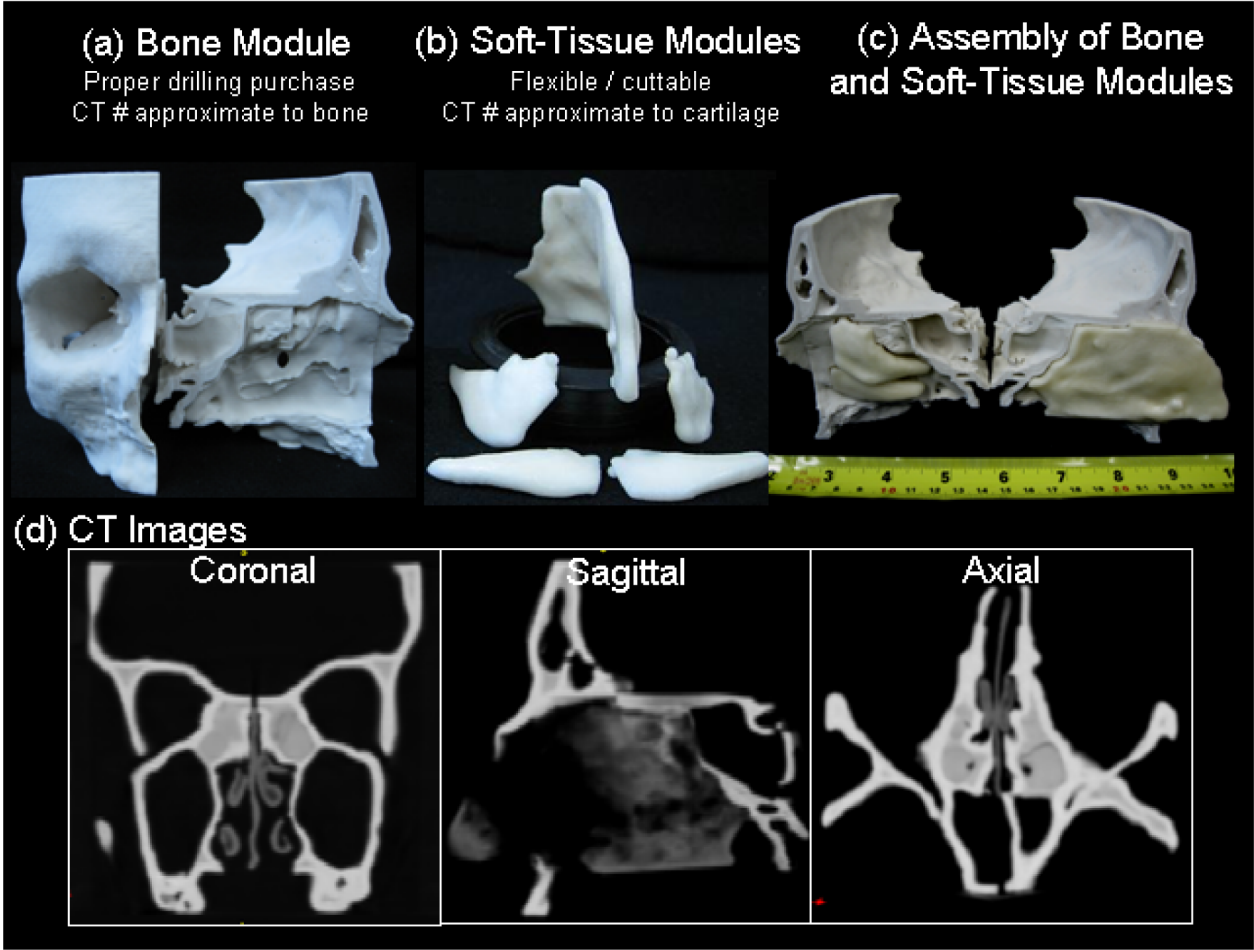
Illustration of a high-fidelity sinus and skull base phantom for surgical simulation. (a) Bone module with realistic sinus structures. (b) Soft-tissue module printed with flexible material that imitates cartilaginous structures. (c) Assembly of bone and soft-tissue with designed joints. (d) CT images of the phantom show appropriate X-ray attenuation number.

The septum and turbinates were categorized as elastic cartilaginous tissue which required a stiff yet flexible material to represent connective tissue. The material selection for soft tissue used a similar approach as described above. After iterating a number of materials and post-processing techniques, ZP-15 plaster powder combined with a post-processing infiltrant elastomeric applied for 30 minutes achieved realistic flexibility. During the post-processing procedure, a ~5–10% expansion was observed in some of the soft tissue components. The final assembly of these components was modified with the aid of surgical tools to reduce the width and length in order to compensate for the expansion. The 3D printer employed for prototyping was a ZPrinter 310 (ZCorp, Burlington MA). The layer thickness is 0.127mm with resolution 300 x 450 dpi. The resulting surgical phantom is shown in Fig 3. Assembly of the modules is incorporated in an anthropomorphic head/torso phantom (Rando, The Phantom Laboratory, Greenwich NY). The cost of rapid prototyping including the bone and soft tissue module is $900 USD.

### Patient-Specific Mandible Templates for Surgical Reconstruction

#### Phantom Requirements

Surgical ablation of infiltrative disease in head and neck, such as maxillectomy and mandibulectomy, requires reconstructive surgery to restore structural and esthetic integrity of the resection site. One such example is reconstruction of the mandible where the objective is to reconstruct the native mandibular contour that will provide for mastication, deglutition as well as provide an adequate airway. Mandibular reconstruction proceeds in two stages: *i*) bone harvest from healthy patient anatomy (e.g. fibula, scapula, iliac crest); and *ii*) grafting of “free-flap” donor tissue to the resection site using mechanical fixation (e.g., titanium plates and screws connecting the harvested bone to adjacent mandible segments) and vascular anastomosis (connecting blood vessels of donor tissue to surrounding anatomy). To restore the natural mandible structure, the conventional approach is to conform the titanium plate to the mandible profile prior to resection, and then connect the donor tissue to the adjacent mandible (post-excision) using this “pre-formed’ titanium plate. In practice, intra-operative mandibular plating is a time consuming process that can take up to 60 minutes for a complex reconstruction [53]. This process is particularly challenging in the setting of invasive disease producing soft-tissue obstruction (e.g., ameloblastoma) or bone fractures (e.g., osteoradionecrosis) within a confined working volume. If such challenges exist, the surgeon’s experience and skill are critical to the reconstruction quality. To overcome such challenges, surgeons have explored alternative approaches with the assistance of 3D models [21, 31–51].

The objective of patient-specific mandible templates is to create a physical 3D model of a patient’s mandible prior to surgery allowing the primary reconstructive surgeon to pre-operatively contour the titanium reconstruction plate freely without any soft-tissue obstructions. To achieve such an objective, the material properties of the template should be rigid enough to withstand the plate blending procedure and be sustainable in a high temperature sterilization process such as an autoclave. The fabrication material selected for template creation is polycarbonate which satisfies all the described criteria.

#### Phantom Design

Using the patient’s previous diagnostic CT scan, the digital processing of mandibular templates begins with importing CT image to the 3D modeling software (Mimics v12.0, Materialise, Ann Arbor MI). Under our hospital protocol of H&N scan, the resolution of the acquired CT image has a pixel size of 0.4 x 0.4 mm^2^ and slice thickness of 2mm. The mandible bone structure is segmented based on voxel intensity (Hounsfield units) using techniques incorporating global thresholding (~550-3000HU), region growing, morphology operation, and smoothing. The fine details of the segmentation are addressed by manual editing on the tri-planer views (i.e., axial, coronal, and sagittal). The segmented mandible is then converted to a mesh model and saved as a Stereo Lithography (STL) file ready for prototyping.

**Fig 4:**
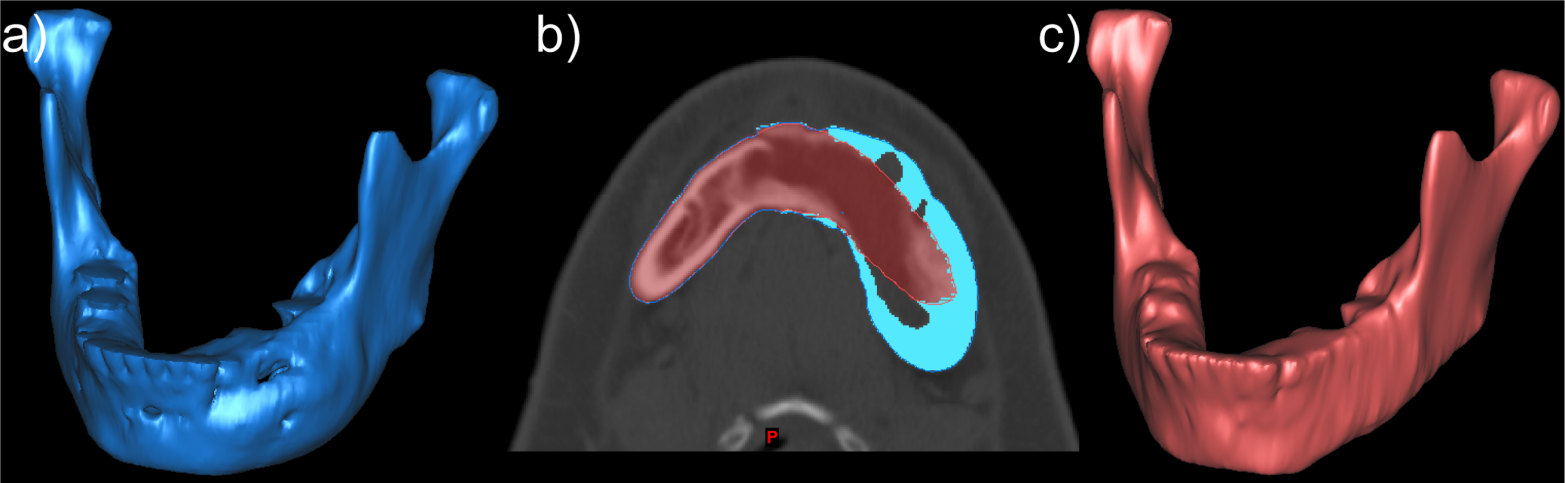
Illustration of computer model created for fabricating a rapid prototype template for mandible reconstruction surgery. (a) Computer model (blue) generated from a diagnostic CT image shows a patient with an aggressive ameloblastoma tumor on the left side of mandible. b) A model of the mandible prior to tumor infiltration (red) is estimated by mirroring the (healthy) right side of the mandible, as shown in an axial image overlayed with the diseased mandible (blue). c) Final computer model of mandible with digital simulation of tumor removal for post-ablation use during mandible reconstruction surgery.

For patients diagnosed with invasive disease distorting the native mandible structure, the mandible is modeled using an alternative approach to recover the normal structure of mandible. This approach is based on a mirroring technique which may be applied when a lesion is located laterally on the mandible. Fig 4 illustrates an ameloblastoma lesion located on the left side of mandible. To recover the normal structure, the right (normal) side of mandible is mirrored to the left (lesion) side as a template for digital editing and removal of the lesion. The mirroring plane is defined at the mid-line of the mandible. The mirror model is then registered to the original mandible to obtain best alignment of the condyle so that the condyle can maintain a neutral geometric relationship with the glenoid fossa. By overlaying the mirror model on the lesion side of mandible, the structure is manually corrected on the tri-planner view and the lesion is digitally removed from the mandible to recover the normal structure (Fig 4C).

#### 3D Fabrication

The rapid prototyping machine employed for fabricating 3D mandible templates is Vantage (Stratasys Inc. Eden Prairie MN), which operates based on fused-deposition modeling (FDM) technology. The material selected for fabricating the mandible template is polycarbonate, which is suitable for an autoclave sterilization process to allow the surgeon to use the template intraoperatively. The design time including CT image import, segmentation and STL file export takes 2 to 4 hours. The 3D printing time for a mandible structure is typically take ~8 hours and dissolving time depends on the complexity of the mandible structure, for example, a mandible fracture would require slightly longer processing time. The cost of in-house fabrication is $110 USD.

## Results

This section covers experimental results for three H&N applications of rapid prototyping described in the previous section: Section 4.1- qualitative assessment of endoscopic phantom; Section 4.2 –surgical simulation using multi-material sinus phantom; and Section 4.3 –clinical implementation of patient-specific mandible templates.

### Phantom for Endoscopist Training and Augmented Reality Development

A qualitative assessment of the endoscopist training phantom was performed by three ESS surgeons using a zero degree rigid endoscope inserted into the nasal passage to examine the realism of the anatomical structures, including the maxillary sinus, cribiform plate and sphenoid sinus. A staff surgeon of otolaryngology with subspecialty in skull base surgery noted that the paranasal morphology of the phantom as similar to human structures and noted that the presentation of the structures was well recognizable. To assess haptic feedback, the surgeon used a tracked pointer probing adjunct anatomy. The surgical sensation of the soft tissue tactile feedback through the instrument was not compliant with mucosal surfaces within a human subject due to the solid fabricating material. The surgeon also noted that the nasal turbinates and ethmoids were excluded in this phantom, making it suitable to simulate an intermediate point in an endoscopic approach to skull base and pituitary tumors.

**Fig 5:**
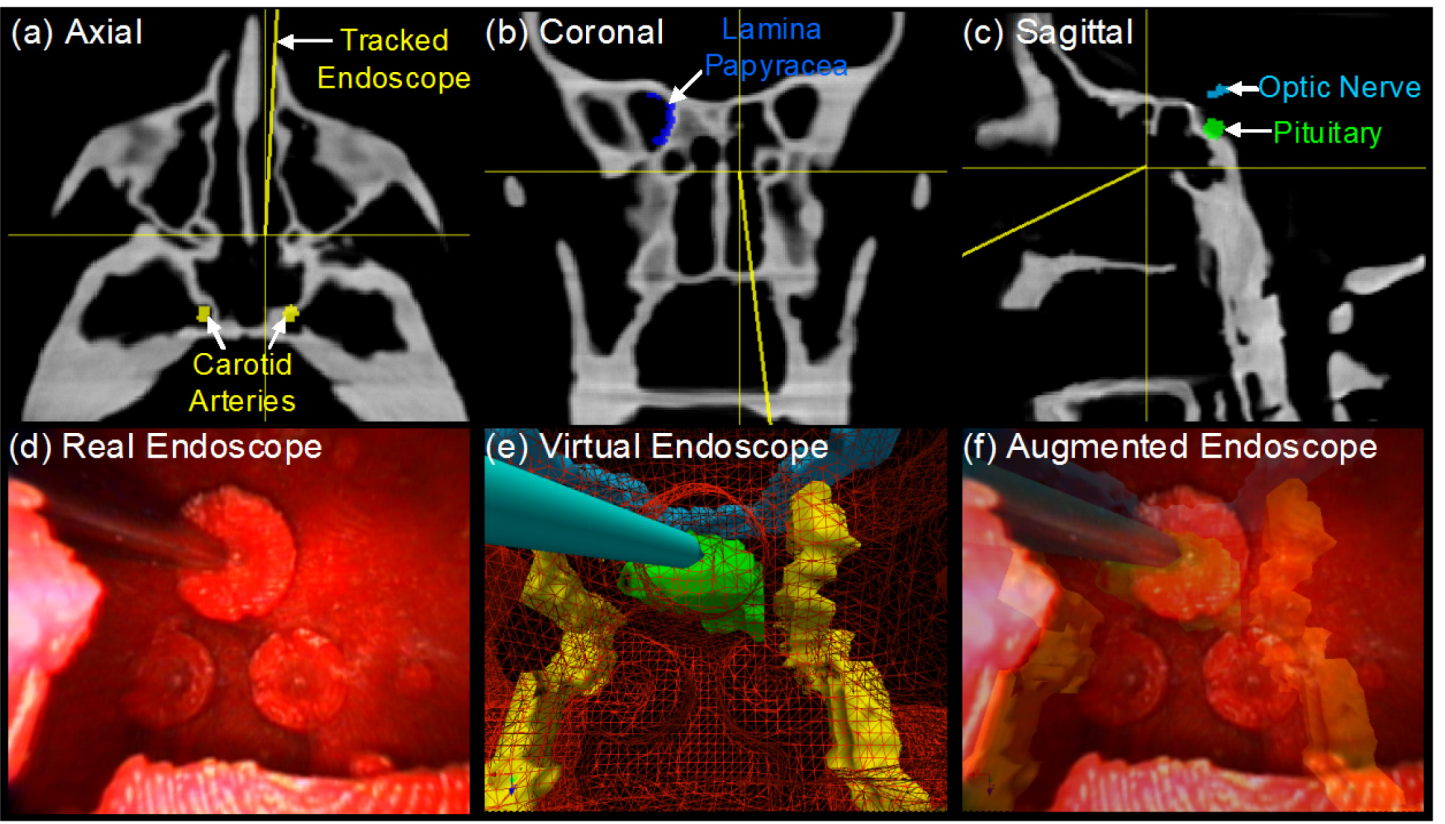
Custom 3D visualization software application illustrating CBCT-endoscopy fusion in the head and neck phantom. (a)-(c) Triplanar CBCT views with surgical contour and tracked tool overlays; (d) endoscopic video with real-time correction of lens distortion; (e) virtual and (f) augmented endoscopic views of semi-transparent CBCT surface renderings (e.g., wiremesh, partial opacity) generated from the perspective of tracked endoscope enable direct visualization of critical structures (e.g., optic nerve, carotid artery) behind surface anatomy.

To facilitate research and development, this phantom was employed to compare the accuracy of manual and automatic methods for image-to-navigation system registration [54]. As well, the phantom was used in studies for endoscope tracking with infrared optical tracker, laryngoscope tracking with electromagnetic tracker, and data fusion of CT and endoscopy images [55, 56]. Fig 5 illustrates registration methods and visualization software for fusion of endoscopy and CBCT [57]. Fig 5A–5C show tri-planar CBCT views of the phantom and the real-time position and orientation of a tracked rigid endoscope. The tri-planar views also include surgical contours of critical anatomical structures (i.e., carotid arteries, optic nerve, lamina papyracea, pituitary) that were delineated in the original cadaveric CT and registered to the phantom CBCT volume. Fig 5D demonstrates the endoscopic view of a surgical pointer within a fiducial marker at the posterior aspect of the sphenoid sinus. Registration of the endoscope position and orientation in the context of intra-operative CBCT enables ‘virtual endoscopic’ views as shown in Fig 5E and 5F which can reveal anatomical structures not directly visible in the endoscopic view. A variety of 3D visualization options regarding endoscopy-CBCT fusion are available including tri-planar oblique CBCT slices at the endoscope tip, semi-transparent surface renderings of CBCT volumes Fig 5E, and augmentation of endoscopic video with CBCT images and planning data (Fig 5F). The endoscopic phantom has been employed in conjunction with virtual and augmented reality visualization technique to provide a teaching environment for junior-level residents to learn endoscope instrument handling and anatomical relationships between radiological and surgical view.

### Advanced Skull Base Surgical Simulation: Image-Guided Surgery with Multi-Material Sinus Phantom

The multi-material sinus phantom for simulating advanced skull base surgery is illustrated in Fig 3. The bone module presents realistic anatomical features, such as the frontal, ethmoid, and sphenoid sinuses, with drilling purchase and CT number (~942 HU) approximating bone. The soft tissue modules (~39 HU) including the septum and nasal turbinates demonstrate flexibility and cut-ability resembling elastic cartilage. The quality assessment of the phantom realism was achieved through image-guided surgical procedures involving ablation of the left/right ethmoid, and clivus. Ablations were conducted by a skull base surgeon using standard endoscopic instruments with the aid of zero degree endoscope. Prior to the procedure, a CBCT baseline scan was acquired for the surgeon to delineate ablation targets using open-source contouring software ITK-SNAP (http://www.itksnap.org/). Intraoperative CBCT images of the phantom were acquired on demand by the surgeon over the course of the procedure. For each guidance scan, the pre-defined targets were registered to the current scan using deformable registration [58]. Contours of the target were overlaid with variable-opacity on the CBCT image to highlight the regions of ablated and residual target. Based on the intraoperative image review, the surgeon decided if any further ablation was required. For the described tasks, the surgeon requested 3, 4 and 3 CBCT scans for left ethmoidectomy, right ethmoidectomy, and clivus ablation, respectively.

The surgeon’s feedback on the phantom for ESS surgery was positive including realistic presentation of nasal cavity, nasal turbinates and nasal septum. During turbinate excision, the surgeon received sensory feedback with turbinectomy scissors, which was similar to real surgery. The surgeon noted that the “fractureness” of cartilaginous sinus cell in the phantom was relatively stiffer than human tissue. Therefore, in the ethmoidectomy procedure, the surgeon used a standard drill (Medtronic Xomed, Jacksonville FL) instead of the typical surgical instrument such as curette, Kerrison rogeur and microdebrider that is normally used for the real patient. In the clivus ablation, the surgeon confirmed the realism of the anatomical structures and the excellent drilling purchase demonstrated in the phantom.

### Clinical Implementation: Patient-Specific Mandible Templates

**Fig 6:**
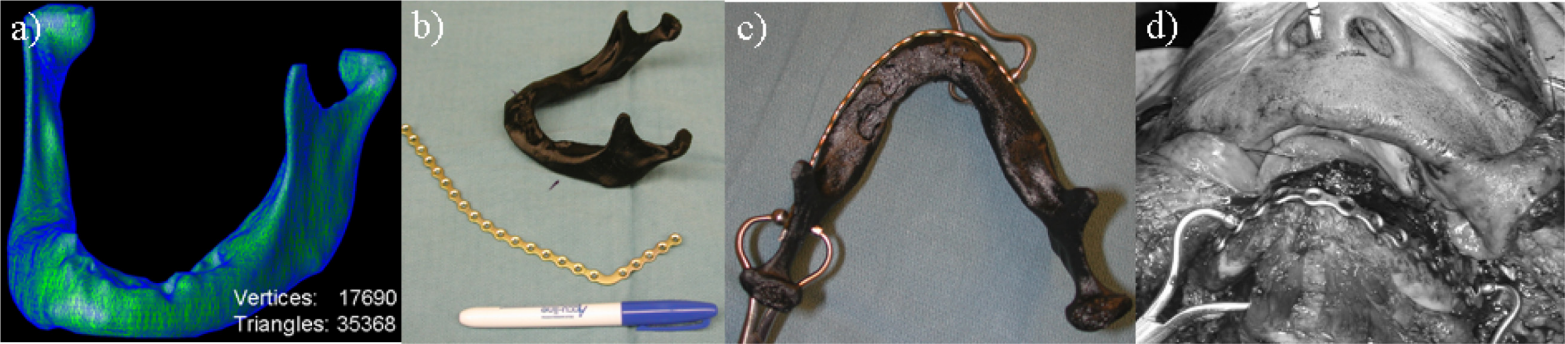
Illustration of patient-specific mandible 3D printing for reconstructive surgery. (a) Mesh wireframe computer model of the mandible. (b) Photograph of the printed mandible with the titanium plate. (c) Pre-operative moulded plate with mandible template, and (d) its use for mandible reconstruction following surgical ablation of a segment of the mandible.

The use of rapid prototyping in mandible reconstruction surgery was investigated through the use of patient-specific mandible templates as illustrated in Fig 6. A clinical trial under institutional research ethics board approval was conducted in 10 mandibulectomy patients to evaluate the utility of patient-specific 3D models and pre-operative plating. All patients received the standard of care procedure including intra-operative contouring of the titanium plate based on anatomy prior to resection if feasible, in comparison to a titanium plate produced pre-operatively based on a physical 3D model of the patient’s anatomy. During the procedure, both preoperative and intra-operative plates were submitted to the two blinded observers (reconstruction surgeons) to judge the plates qualitatively in terms of geometry and conformance to the reconstruction site. The qualitative judgment conducted in the form of a questionnaire included the questions: 1) How well does the plate match the contour of the mandible? 2) Evaluate the percentage of surface area contact between the plate and mandible; 3) Which plate is the overall best match? In question 1, a five point Likert scale (1–5) was employed to evaluate the performance of the plate. The median score of intra-operative plate was 4 with interquartile range (IQR) 1, while the preoperative score was 4 with IQR 0. There were no statistically significant differences between intra-operative and pre-operative plating from two observers (p = 0.38, Wilcoxon signed-rank test). Regarding the second question, a 10 point Likert scale employed, the median score and IQR of the percentage of surface contact for intra-operative and preoperative plates were both 8 (70% to 80% surface contact) and 1.25. The Wilcoxon signed-rank test yielded a p-value of 0.14 (p>0.05), therefore no statistical significant differences were observed. Over 10 patients, observer 1 scored 6 out of 9 pre-operative plates as the best plate for the surgery. Observer 2 evaluated 6 out of 9 pre-operative plates as outperforming the intra-operative plate. In only two cases did the choice of optimal plate differ between the observers. The intra-operative plating was not available in one of the cases because of the severity of the ameloblastoma tumor extending laterally and preventing contouring, therefore, the pre-operative plate was the only viable option for the surgery. The average time spent for intra-operative and pre-operative contouring was 833 and 867 seconds, respectively. The two contouring procedures differ in their impact on clinical workflow, with no additional OR time required for pre-operative plating. 7 out of 10 preoperative plates were selected for the final reconstructive plate in the surgery. 3 out of 10 intra-operative plates were selected for a final reconstructive plate for the surgery.

## Discussion

This study elucidates techniques for the fabrication of 3D phantoms for head and neck surgery based on advanced rapid prototyping technology. Such phantoms offer great potential as a tool for surgical education, training residents and treatment planning. However, the limited statistical analyses from these preliminary investigations are currently inadequate to determine the influence of the phantoms on clinical practice and a more robust prospective clinical trial will be required to assess the clinical impact of such phantoms. Respecting the lengthy learning process in acquiring technical skills in surgery, the proposed phantoms can be employed as adjunct tools in residency training. They allow resident surgeons to practice psychomotor skills while maneuvering endoscopic instruments within a confined, realistic environment with a complex, yet critical, anatomy. Such navigation tool skills can be acquired independently without exposing a patient to the inherent risks of having a novice surgeon performing a surgical resection. Additionally, we demonstrated that the endoscopic sinus and skull base phantoms can be used as an alternative test-bed, and may replace the need for cadaveric specimens when developing a novel surgical guidance technology. The ESS phantom allows for verification and validation of the concept, design, feasibility and actual surgical application. The role of 3D phantoms providing such verification and validation was very important for advancing each step in the development, providing proof of technology principle at each intermediate procedure. In this context, the use of phantoms is preferable in that it avoids considerations associated with biological specimens such as resource availability, biological hazards, storage facilities, and ethical issues. High-fidelity phantoms generated using digital segmentation techniques also enables repeatable studies with customized anatomical scenarios that are not feasible when using cadavers.

The first phantom described in the paper demonstrated the role of realistic endoscopic phantoms in the development of novel augmented reality and tool tracking systems. In addition to providing realistic anatomical structures, the custom fiducial markers provide the capability to perform quantitative analysis of registration performance (e.g., target registration error and fiducial registration error). Expert feedback on the realism of this phantom to date has only included three surgeons. In future, feedback from a larger cohort of experienced surgeons and junior residents will be required to demonstrate the benefits for endoscopic skill acquisition. We expect that the models likely provide greater benefit to resident or junior surgeons still on the steep part of the learning curve.

The second phantom for surgical simulation makes use of two materials to mimic bone and soft-tissue to provide a realistic 3D phantom for training and simulation of ablative surgical procedures. Such fabrication techniques will enable surgeons to customize their own phantom or generate a patient-specific model for rehearsal purposes prior to surgery. The fabrication technique also potentially allows the creation of a digital/physical atlas that includes models presenting anatomical variations and disease to meet the need of surgeons in training, simulation, and performance evaluation. Additionally, the surgical simulation phantom provides an evaluation platform for critical analysis of surgical performance in the complex tasks performed under endoscopic or open approaches to the rich 3D architecture of the paranasal sinuses and skull base. The capability of reproducing identical phantom creates a controllable environment for repeat testing that enables analysis of the development of novel technologies such as C-arm CBCT and experimental surgical techniques. The ability to create identical tasks for a given number of surgeons permits quantitative evaluation of improvements in surgical efficacy and safety.

The patient-specific rapid prototyping 3D mandible facilitates contouring mandibular reconstruction preoperatively. While this study subjectively compared preoperative plates with traditional intraoperative plates in a relatively small case series, it nevertheless demonstrates a proof of concept that a preoperatively contoured plate may provide equivalent or better conformance than conventional intraoperative plates. The most tangible example of the benefits of preoperative contouring are when the neoplastic process extends on the buccal aspect of the mandible thereby preventing contouring an accurate intraoperative mandibular reconstruction plate. In these circumstances, the prototyping template can be applied to recover the normal structure and provide cosmetic improvement. This technique also has a significant potential to shorten the length of OR time for the surgical team and consequently reduce operative cost in the hospital. Similarly, the novel modeling technique of digitally removing gross pathology from the mandible prior to 3D fabrication enables template bending without the need for a time intensive external fixation device during intraoperative plating. The analysis from this initial case series presents a preliminary finding. While the sample size is limited and therefore lacks sufficient statistical inference it nevertheless demonstrates a proof of concept and provides motivation for a larger prospective study comparing the presented preoperative plating technique to traditional intraoperative techniques. Clinical evaluation is ongoing to validate the benefits of patient-specific mandible modeling for improved patient outcomes.

## Conclusion

The application of rapid prototyping technology in head and neck surgery is demonstrated in the development of three custom anatomical models: i) a mono-material paranasal sinus phantom; ii) a multi-material skull base phantom for surgical ablation simulation; and iii) patient-specific mandible templates. Each model illustrates the potential benefit for surgical education, procedure simulation and clinical practice. The applicability of model design and fabrication techniques here is not limited to otolaryngology, however, it is highly adaptable for a variety of surgical applications.

## Supporting Information

S1 File Head and Neck Phantoms STL files. (ZIP)
